# Feasibility, acceptability, and utility of a nurse-led survivorship program for people with metastatic melanoma (MELCARE)

**DOI:** 10.1007/s00520-022-07360-4

**Published:** 2022-09-22

**Authors:** Julia Lai-Kwon, Brooke Kelly, Sarah Lane, Rebecca Biviano, Iris Bartula, Frank Brennan, Ingrid Kivikoski, Jake Thompson, Haryana M. Dhillon, Alexander Menzies, Georgina V. Long

**Affiliations:** 1 Melanoma Institute Australia, Sydney, Australia; 2 Melanoma Patients Australia, Varsity Lakes, Australia; 3 Faculty of Medicine and Health, The University of Sydney, Sydney, Australia; 4 Consumer Representative, Sydney, Australia; 5 Consumer Representative, Brisbane, Australia; 6 Centre for Medical Psychology & Evidence-Based Decision-Making, School of Psychology, Faculty of Science, University of Sydney, Sydney, Australia; 7 Psycho-Oncology Cooperative Group, School of Psychology, Faculty of Science, University of Sydney, Sydney, Australia; 8 Royal North Shore Hospital, Sydney, Australia; 9 Mater Hospital, Sydney, Australia

**Keywords:** Melanoma, Survivorship, Nursing, Telehealth, Quality of life

## Abstract

**Purpose:**

Immune checkpoint inhibitors (ICIs) and targeted therapy (TT) have improved the survival of people with metastatic melanoma. We assessed the feasibility, acceptability, and utility of a novel model of nurse-led, telehealth-delivered survivorship care (MELCARE) for this survivor group.

**Methods:**

People ≥ 18 years diagnosed with unresectable stage III or stage IV melanoma who were ≥ 6 months post initiation of ICI/TT with a radiological response suggestive of a long-term response to ICI/TT were recruited from a specialist melanoma centre in Australia. All participants received MELCARE, a nurse-led survivorship program involving two telehealth consultations 3 months apart, needs assessment using the Distress Thermometer (DT) and Problem List, and creation of a survivorship care plan. Feasibility, acceptability, and utility were assessed using rates of consent and study completion, time taken to complete each component of MELCARE, the Acceptability of Intervention Measure (AIM), and a customised utility survey.

**Results:**

31/54 (57%) people consented. Participants were male (21, 68%), with a median age of 67 (range: 46–82). Eleven (35%) were receiving/had received ipilimumab and nivolumab and 27 (87%) had ceased treatment. Feasibility was demonstrated with 97% completing MELCARE. Utility was demonstrated on a customised survey and supported by a reduction in the mean DT score (initial: 5.6, SD: 2.9; follow-up: 1.5, SD: 1.2). Acceptability was demonstrated on 3/4 AIM items.

**Conclusion:**

MELCARE was feasible and acceptable with high levels of utility. However, the consent rate was 57% indicating some people do not require support. Future studies should consider MELCARE’s optimal timing, resourcing, and cost-effectiveness.

**Supplementary Information:**

The online version contains supplementary material available at 10.1007/s00520-022-07360-4.

## Background

The prognosis of people with metastatic melanoma was historically poor, with a 5-year overall survival (OS) of approximately 7% [1]. The advent of immune checkpoint inhibitor therapy (ICI) and targeted therapy (TT) has significantly improved the survival of a subgroup of people with metastatic melanoma with around 50% achieving durable disease control [2–4].

However, this group of long-term survivors may experience unique physical, psychological, social, and functional concerns and unmet needs not routinely screened for or addressed in clinical encounters [5–10]. They include chronic ICI and TT-related toxicities, such as rashes, myalgias, fatigue, and diarrhoea [5–8]. Psychological issues, including difficulties dealing with uncertainty, an inability to plan for the future, fear of recurrence or progression, and anxiety and depression have also been reported [5, 7, 11, 12]. Objective neurocognitive impairment has been observed several years after immunotherapy cessation in two single centre studies [13, 14]. Social, financial, and functional concerns may include difficulties undertaking domestic tasks, recreational activities, and planning or taking holidays [5, 12], difficulty paying for transport/parking or accommodation [12], or accessing insurance payouts [5].

Currently, there are no survivorship programs specifically designed to support people with metastatic melanoma who are long-term responders to ICI/TT. Survivorship programs are typically aimed at people with cancer treated with curative intent and may not meet the specific needs of long-term responders with metastatic malignancies [15, 16].

Nurse-led survivorship care has been shown in other tumour types to be safe, effective, holistic, and can support care coordination, self-management, and behaviour change, and produce high levels of personal satisfaction [17–23]. It is similarly efficacious in managing physical and psychosocial outcomes, with additional economic benefits and reduced use of healthcare resources compared to specialist-led care [24]. Nurse-led care is also more acceptable to people, increases satisfaction due to increased convenience and shorter waiting times, and improves overall quality of life [21, 25]. Specialist melanoma nurses have the necessary experience and skills to holistically manage the complexities of a long-term responder’s survivorship care, positioning them to co-design and deliver an effective survivorship program. Furthermore, delivering nurse-led care to people with metastatic melanoma via telephone has been shown to be feasible and acceptable, indicating that people are willing to engage with remotely delivered support services [26].

We designed a novel model of nurse-led, telehealth survivorship care for people with metastatic melanoma who are likely to be long-term responders to ICI/TT (MELCARE). We conducted a pilot study to assess the feasibility, acceptability, and utility of MELCARE.

## Methods

This study was conducted at the Melanoma Institute Australia (MIA). Ethics approval was obtained from Royal Prince Alfred Hospital Ethics Review Committee (protocol number: X21-0276). All participants provided written informed consent.

### Participants

Potential participants were identified through weekly review of melanoma clinic lists. People aged ≥ 18 years, with histologically confirmed AJCC 8^th^ edition unresectable stage III or stage IV melanoma, who were likely long-term responders to ICI or TT based on computed tomography (CT) and/or positron emission technology (PET) criteria, proficient in English, and able to participate in a telephone consultation and complete electronic surveys were eligible.

The identification of potential long-term responders was informed by several studies examining long-term outcomes of people based on their radiological response to therapy [3, 27–30]. If ICIs were their most recent therapy, participants needed to be ≥ 6 months post-initiation of their current line of ICI therapy and had a complete response (CR) on CT scan or be ≥ 1-year post-initiation of their current line of ICI therapy and had a complete metabolic response (CMR) on PET with a CR or partial response (PR) on CT scan. If TT was the most recent therapy, participants needed to be ≥ 2 years post-initiation of their current line of TT and had a CR on CT scan.

### Recruitment

Eligible people were contacted by telephone by JLK/SL/RB to discuss the study. Interested people were emailed an electronic consent form on Research Electronic Data Capture (REDCap, version 10.3.4, Vanderbilt University). Reasons for declining participation were noted. Baseline demographic and clinical characteristics were extracted from the medical record. Participants were contacted by BK to book their initial consultation.

### MELCARE intervention

**Fig. 1:**
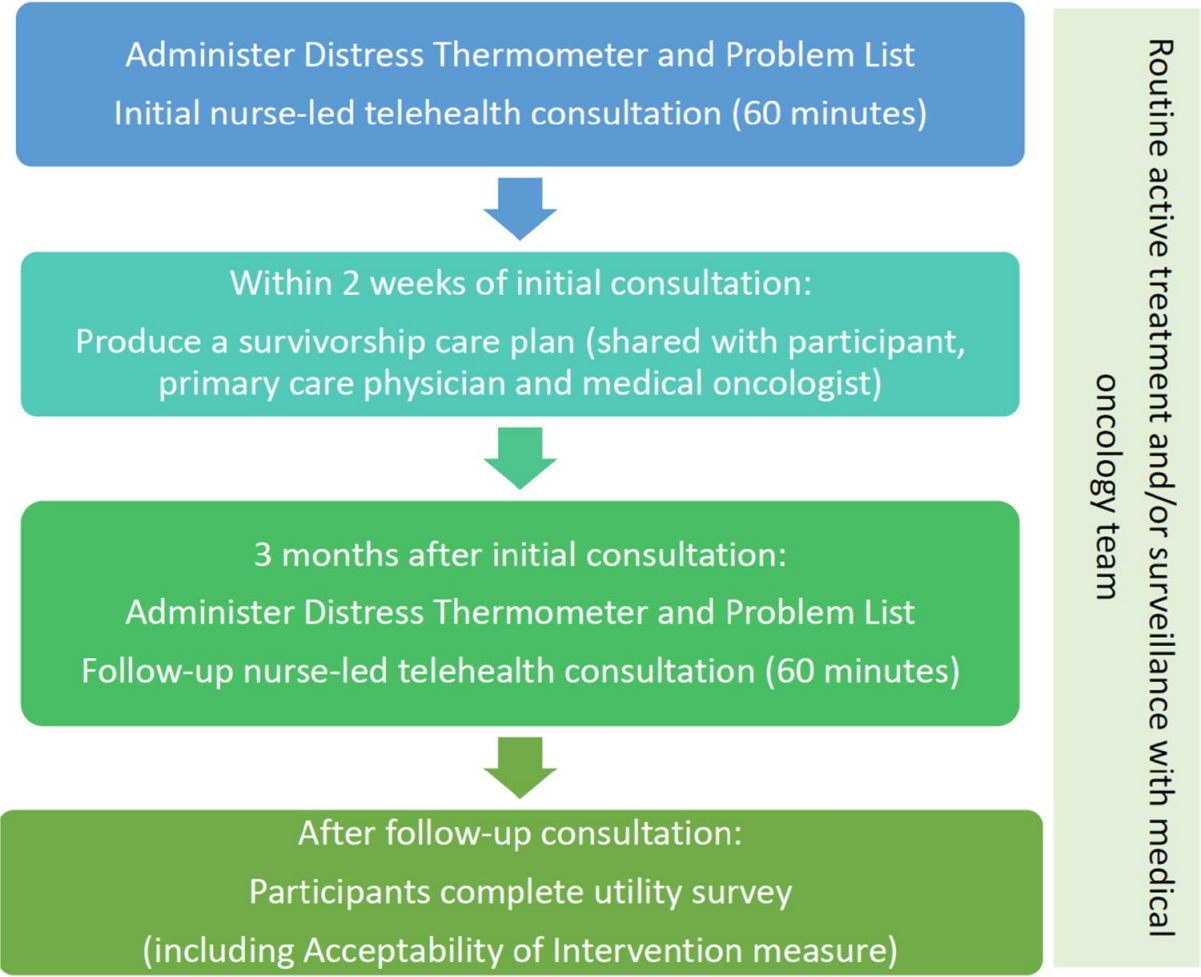
MELCARE intervention

MELCARE was designed by a multidisciplinary team of health care professionals and consumers from MIA and Melanoma Patients Australia (MPA). It consisted of two, 1 h, melanoma nurse-led consultations conducted via telephone 3 months apart. Each consultation included a holistic needs assessment using standardised patient-reported outcome measures (PROMs). A personalised survivorship care plan (SCP) was also created following the initial consultation (Fig. 1). The intervention was delivered by BK, a registered oncology nurse with experience in supporting people with melanoma.

The initial consultation involved an assessment of the patient’s concerns and unmet needs using several PROMs: the National Comprehensive Cancer Network (NCCN) Distress Thermometer (DT) and Problem List for Patients (Version 2.2018), with higher scores on the DT indicating a higher level of distress and a score of ≥ 4 indicating a clinically relevant level of distress warranting further investigation [31–33]; and a bespoke Additional Problem List designed by the authors including more specific physical, psychological, social, and functional concerns and unmet needs faced by this population based on prior research [5–7, 9, 13] (Supplementary text 1). PROMs were administered verbally by BK and scores recorded. This was a pragmatic decision to minimise the administrative workload of sending, receiving, and checking PROMs prior to the consultation. This was followed by a discussion of the issues identified, and self-management strategies, internet resources, and referrals that could be arranged by their melanoma medical oncology team or primary care physician (PCP). To standardise the advice provided during the consultation, an expected issues list (Supplementary text 2) was developed by a multidisciplinary group of MIA clinicians and researchers with knowledge of both evidence-based strategies, local resources, and services. The issues list catalogued common issues and provided standardised advice, self-management strategies, internet resources, and possible referrals for each. The top three priorities identified by the participant were then summarised in the SCP (Supplementary text 3) and emailed to the participant, their medical oncology team, and PCP. All participants’ PCPs were contacted via phone to make them aware of the SCP.

The follow-up consultation involved a repeated administration of the DT, Problem List, and Additional Problem List, followed by a further discussion of previously identified issues.

Throughout the study, participants continued to receive standard of care treatment and/or surveillance.

### Feasibility, acceptability, and utility data

Feasibility data was collected during the study, including time taken to book and conduct each consultation and prepare the SCP, and the percentage of participants who completed the initial and follow up consultations, the percentage of participants who had a SCP produced within two weeks and sent to their PCP within one month of the initial consultation.

Acceptability data included the percentage of eligible people who signed consent, reasons for declining participation, and participant scores on the Acceptability of Intervention Measure (AIM) at study completion. This is a 4-item survey measuring whether a given treatment/service meets the participant’s approval is appealing to the participant and is liked and welcomed by the participant [34]. Acceptability was defined as being ≥ 70% of eligible people signing consent and ≥ 70% of participants responding ‘completely agree’ or ‘agree’ to each item on the AIM measure.

Utility data was collected from participants. Participants were asked to report whether they a) referred to their SCP in the last 3 months; b) used any information detailed within the SCP; and c) seen their PCP and discussed/ referred to their SCP during the consultation. Participants also completed a customised, 22-question, electronic survey after the follow-up consultation assessing the overall utility of MELCARE (Supplementary text 4).

### Statistical analysis

The sample size for this pilot study was determined by available nursing time and resources. We aimed to recruit 30 participants over three months.

Participant demographic and clinical characteristics, feasibility and acceptability endpoints, and utility questionnaires responses were summarised using descriptive statistics. Time-based feasibility assessments will be presented as a mean duration of time. Analyses were conducted using Microsoft Excel.

## Results

### Participant characteristics

**Fig. 2:**
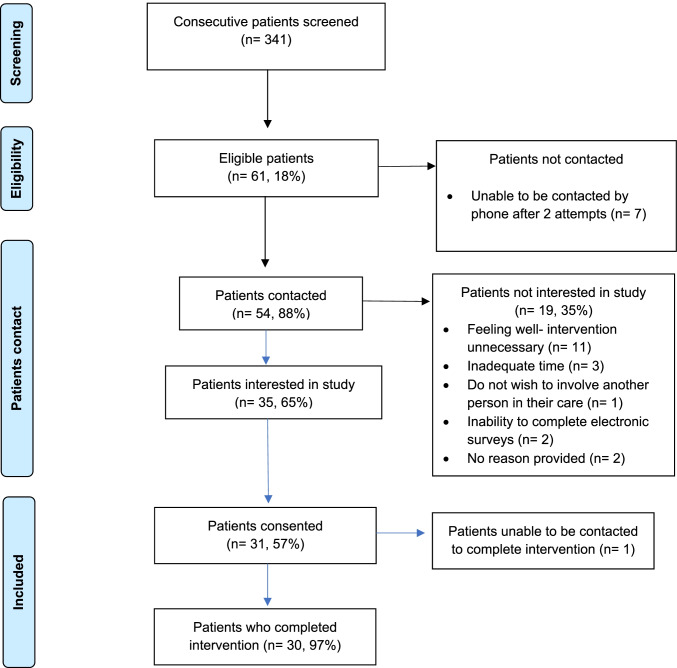
MELCARE recruitment

From 11 to 26 October 2021, 341 consecutive people were screened (Fig. 2); 61 (18%) were eligible, and 54 (88%) were contacted to discuss the study. Of these, 35 (65%) expressed interest and 31 (57%) consented. Of the 19 (35%) who did not express interest, reasons included feeling well making the intervention unnecessary (*n* = 11), inadequate time (*n* = 3), not wishing to involve another person in their care (*n* = 1), inability to complete electronic surveys (*n* = 2), and two did not provide a reason.

MELCARE was completed by 30/31 (97%) participants. A single participant consented but could not be contacted to complete the intervention. Baseline demographic and clinical characteristics are shown in Table 1; the median age was 67 years (range: 46–82) and 21 (68%) were male. Twenty-eight (90%) had American Joint Committee on Cancer (AJCC) 8^th^ edition stage IV disease and 3 (10%) had unresectable stage III disease. The mean time from diagnosis of advanced melanoma to consent was 3.6 years (standard deviation, SD: 1.8 years), and 28 (90%) had received one line of therapy at the time of study enrolment.

**Table 1:** Baseline demographic and clinical characteristics

		N	%
Sex	Male	21	68
	Female	10	32
Age	Median (range)	67 (46–82)	
AJCC stage	Unresectable III	3	10
	IV (M1a)	1	3
	IV (M1b)	13	42
	IV (M1c)	12	39
	IV (M1d)	2	6
BRAF	Wild type	18	58
	Mutant	12	39
	Not available	1	3
LDH	Normal	16	52
	Elevated	5	16
	Not available	10	32
Time from diagnosis of metastatic melanoma to consent (years)	Mean (standard deviation)	3.6 (1.8)	
Currently on treatment?	Yes	4	13
	No	27	87
If on treatment, current treatment regimen	Ipilimumab and nivolumab, then maintenance nivolumab	3	10
	Pembrolizumab and lenvatinib	1	3
If off treatment, most recent treatment regimen	Ipilimumab and nivolumab, then maintenance nivolumab	8	26
	Pembrolizumab	4	13
	Nivolumab ± anti-LAG3	4	13
	Nivolumab	3	10
	Nivolumab and anti-LAG3	2	6
	Ipilimumab and pembrolizumab	2	6
	Pembrolizumab and anti-TIGIT	1	3
	Pembrolizumab + / − anti-GITR	1	3
	Pembrolizumab and anti-GITR	1	3
	Probody (BMS-986249) and nivolumab	1	3
Reason for discontinuation	Completed 2 years	8	26
	Toxicity	17	55
	Patient choice	2	6

Four participants were receiving active treatment; the most common treatment regimen was ipilimumab and nivolumab followed by maintenance nivolumab (3/4, 75%). Twenty-seven (87%) were no longer receiving treatment at the time of enrolment; the main reason for discontinuation of therapy was toxicity (17, 55%). The most recent treatment regimens were ipilimumab and nivolumab followed by maintenance nivolumab (8, 30%) and single-agent nivolumab or pembrolizumab (7, 26%).

### Feasibility

30/31 (97%) participants received an initial and follow-up consultation. The mean time to arrange and book the initial and follow-up consultations were 6 (SD: 2.3) and 7 min (SD: 3.7) respectively. The mean time to conduct the initial consultation was 35 min (SD: 9) and the follow-up consultation was 29 min (SD: 10). The mean time to prepare the SCP was 19 min (SD: 5). The mean total nursing time spent on delivering MELCARE was 96 min (SD: 15).

### Acceptability

**Fig. 3:**
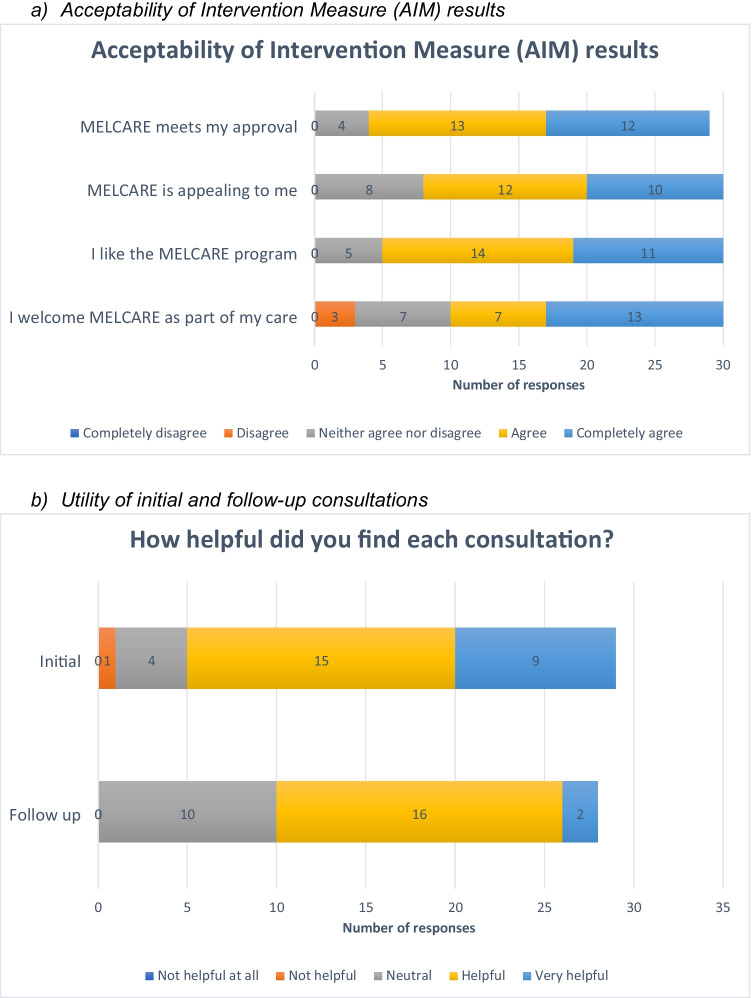
Key acceptability and utility results

Recruitment to the intervention was completed in 15 days. 31/54 (57%) eligible participants signed consent. On the AIM, 25/29 (86%) stated MELCARE met their approval (completely agree/ agree), 22/30 (73%) stated it was appealing to them, 25/30 (83%) stated they liked the program, and 20/30 (67%) stated they would welcome it as part of their care (Fig. 3a).

Participants who were < 2 years from their diagnosis of advanced melanoma were more likely to rate items on the AIM as completely agree/agree compared to those who were ≥ 2 years from their diagnosis. 6/6 (100%) participants < 2 years from their diagnosis stated that MELCARE met their approval compared to 19/24 (79.1%) participants ≥ 2 years from their diagnosis. Similarly, 5/6 (83.4%) participants < 2 years from their diagnosis would welcome MELCARE as part of their care compared to 15/24 (62.5%) participants ≥ 2 years from their diagnosis.

### Utility

There was a reduction in the mean DT score and mean number of problems identified on the Problem List between the initial and follow up consultations [Initial DT score: 5.6 (SD: 2.9), follow up: 1.5 (SD: 1.2); initial Problem list: 2.6 (SD: 1.8), follow up: 0.8 (SD: 1.1)]. The mean number of problems identified on the initial Additional Problem List was relatively stable [initial: 0.1 (SD: 0.3), follow up: 0.6 (SD:1.4)].

At the initial consultation, the three most common issues reported on the Problem List were fatigue (19/30, 63%), sleep (17/30, 57%), and dry/itchy skin (16/30, 53%). At the follow-up consultation, they were fears (8, 27%), fatigue (7/30, 23%), and sleep (6, 20%). At the initial consultation, the three most common issues reported on the Additional Problem List were fear of melanoma recurrence/ progression (18/29, 62%), anxiety around the time of scans (15/29, 52%), and joint aches/pains (12/29, 41%). The issues were the same at the follow up consultation (anxiety around the time of scans (13/30, 43%), fear of melanoma recurrence/progression (10/30, 33%), and joint aches/pains (8/30, 27%)).

Figure 3b shows the proportion of patients who found both consultations helpful. 29/29 (100%) thought the initial consultation was sufficient in length. The three most useful aspects of the initial consultation were discussing side effects (17 responses, 59%), discussing emotions (16, 55%), and being provided with an SCP to share with their PCP and other healthcare professionals (12, 41.4%). The three most useful aspects of the follow-up consultation were discussing emotions (11, 38%), discussing side effects (10, 35%), and reviewing personal goals (8, 28%). Supplementary Table 1 shows suggestions for improving both consultations.

Participants felt that the SCP was useful for providing written advice about managing their health (12, 41.4%), setting personal goals for their ongoing care (9, 31%), and for providing websites/podcast recommendations (8, 27.6%) and general information regarding skin checks (8, 27.6%). Suggested improvements included providing more details about their melanoma diagnosis and treatment (6, 27.3%) and more detail regarding referrals their PCP could arrange (5, 22.7%).

27/29 (93%) self-reported reading their SCP, with 23/26 (88%) rating it as easy or very easy to understand. 10/29 (35%) self-reported discussing their SCP with their PCP. Of those who had not, 9/19 (47%) were planning on doing so in the next three months, 6/19 (32%) did not feel they required any help from their PCP, 3/19 (16%) did not feel their PCP was able to assist them, and 1/19 (5%) preferred to discuss their SCP with their usual care team.

22/30 (73%) thought two consultations were sufficient, with only four (13%) suggesting more than two consultations were required. There was no difference in the number of preferred consultations in participants who were < 2 years compared to those who were ≥ 2 years from their diagnosis of metastatic melanoma. However, participants with an initial DT score ≥ 4 were more likely to prefer two appointments compared to those with an initial DT score < 4 (4/20, 20% vs 0/10, 0%). 28/28 (100%) thought sufficient time was available to discuss their issues.

22/28 (79%) stated MELCARE improved their overall satisfaction with their care. 16/28 (57%) stated it helped them manage issues not discussed during their medical oncology appointments, such as treatment-related side effects (9, 56%), emotional issues (5, 31%), and information regarding skin surveillance for primary melanoma (4, 25%). 28/28 (100%) would recommend it to other people with metastatic melanoma.

## Discussion

The concept of ‘metastatic survivorship’ is increasingly recognised as the prognosis of people with metastatic cancers improves [35–38]. Access to novel therapies, such as ICI and TT, has significantly improved the prognosis of a subset of people with certain metastatic cancers, such as metastatic melanoma, non-small cell lung cancer, and renal cell carcinoma [35–40]. Many now experience metastatic cancer as a chronic, complex illness with a variable disease trajectory (including periods with and without active treatment, and with and without disease control) and prognostic uncertainty. Models of survivorship care must recognise the complexity of supportive care needs in this patient group, including management of acute and chronic toxicities, and psychosocial and practical needs.

MELCARE is the first study to pilot a model of survivorship care in people with a metastatic malignancy who are long-term responders to ICI or TT. MELCARE has demonstrated preliminary evidence of its feasibility and utility, and partial evidence of its acceptability. Whilst this pilot study did not assess the efficacy of the intervention, a reduction in mean DT scores from a clinically significant level of distress (DT score ≥ 4) to below a clinically significant level of distress was noted. While this is only hypothesis generating, it does indicate that MELCARE warrants further exploration in a larger trial adequately powered to assess efficacy. MELCARE was successfully delivered during the COVID-19 pandemic, demonstrating that survivorship care delivered via telehealth by a centralised nurse is feasible and can improve accessibility and equity of access to survivorship care.

The study also identified the key concerns and unmet needs of this survivor group, with fatigue, sleep issues, fear of cancer recurrence, and dry/itchy skin frequently reported at both the initial and follow-up consultations. These issues are consistent with previous studies examining the physical, psychological, and social concerns and unmet needs of people with metastatic melanoma who are long-term survivors of ICI/TT [5–10]. This highlights the importance of systematically screening people for these issues and identifies priorities for supportive care interventions for this population, with several interventions already under investigation. This includes a stepped care program to manage fear of cancer recurrence/progression in people with stage IV melanoma [41] and an exercise program for people with ICI-related fatigue [42]. The Additional Problem List identified several issues unique to this patient group. Whilst this list has not been validated, the high proportion of participants reporting issues provides preliminary evidence of its content validity which can be evaluated in future studies and highlights the need for specific PROMs for people with metastatic melanoma.

MELCARE has several limitations. Firstly, it was resource-intensive with an average of 96 min of nursing time per participant. This is significant in the context of limited specialist melanoma nurses and does not include the time or cost of interventions to manage any issues raised. To minimise the nursing administrative burden, PROMs were assessed verbally during the consultation, but this may have affected their validity and increased the nursing time required. The study highlights the need for greater funding for survivorship nurses and supportive care services (such as psychology and social work) to manage issues raised. Future studies will need to assess longer-term patient outcomes, whether these outweigh the upfront costs associated with implementing MELCARE and whether MELCARE is ultimately cost effective. Secondly, low levels of PCP engagement were noted with only 35% of participants discussing their SCP with their PCP. This may be partly due to limited access to PCPs due to the COVID-19 pandemic. However, 16% felt they PCP was unable to assist with any issues raised. This indicates the importance of survivorship-focused education for PCPs regarding the needs of this emerging survivor group and the importance of developing shared models of survivorship care. Thirdly, part of the criteria for acceptability was not met with only 57% of eligible participants consented. The most common reason for declining the study was feeling well making the intervention unnecessary. Given the mean time from diagnosis of advanced disease to consent was 3.6 years, this may suggest that people may benefit from the intervention earlier in their disease course. This was supported by the observation that participants earlier in their disease trajectory (< 2 years from their diagnosis) were more likely to rate AIM items as ‘completely agree’ or ‘agree’ compared to those later in their disease trajectory (≥ 2 years from their diagnosis), although the study was not powered to statistically investigate differences between the two groups. Furthermore, those with a baseline DT score ≥ 4 were more likely to want more than 2 consultations, indicating that they may benefit most from MELCARE. These considerations highlight those subgroups most likely to benefit from the intervention. It is also possible that those who elected to participate in MELCARE may be more inclined to rate the intervention positively and this should be taken into consideration when interpreting these results. Finally, this study was conducted in a single centre in an English-speaking population. Future studies should aim to assess MELCARE in a broader population from a range of facilities and culturally and linguistically diverse backgrounds.

Future studies should incorporate participant suggestions for improvements to MELCARE, such integrating video calls or conducting the consultations in person. They should also assess the utility of MELCARE to healthcare professionals, such as medical oncologists and PCPs. For PCPs, it would be interesting to measure the impact of MELCARE on their management of comorbid chronic diseases and preventative health activities. MELCARE also lays the foundation for future survivorship research for people with metastatic cancer. Whilst this intervention was specifically aimed at long-term responders to ICI/TT who may be cured of their melanoma, the management of chronic treatment-related toxicities and provision of psychosocial support remains a priority in people with indolent disease biology but ultimately a limited life expectancy. Survivorship programs should acknowledge these prognostic challenges upfront and include protocols for transitioning back to acute care in the event of progressive disease.

## Conclusion

This is the first study to pilot a nurse-led telehealth model of survivorship care in people with a metastatic malignancy who are long-term responders to ICI/TT. MELCARE was feasible with high levels of participant utility, although only 57% of eligible people consented. This addresses a critical unmet supportive care need in this emerging survivor group. Future studies should measure its impact on patient outcomes and explore the optimal timing, resourcing, and cost-effectiveness of MELCARE.

## Supplementary Information

Below is the link to the electronic supplementary material.

Supplementary file1 (DOCX 13 KB)

Supplementary file2 (DOCX 21 KB)

Supplementary file3 (PDF 466 KB)

Supplementary file4 (PDF 153 KB)

Supplementary file5 (DOCX 35 KB)

## Data Availability

De-identified participant data underlying the results reported in this article will be shared. Data will be available immediately following publication with no end date. Investigators who propose use of the data that has been approved by an independent review committee will be granted access to the data to achieve the aims of the approved proposal. Proposals should be directed to Julia.Lai-Kwon@petermac.org.
