# Supplementary material for: Feasibility, acceptability, and utility of a nurse-led survivorship program for people with metastatic melanoma (MELCARE)
Source: Support Care Cancer. 2022 Sep 22;30(11):9587–96. doi: 10.1007/s00520-022-07360-4 (PMC9492451; doi:10.1007/s00520-022-07360-4)
Supplement: Supplementary file 1 — Supplementary file1 (DOCX 13 KB) [file 520_2022_7360_MOESM1_ESM.docx]

**Supplementary Table 1:** *Participant suggestions for improving the initial and follow-up consultation*

|  | Initial consultation  N (%) | Follow-up consultation  N (%) |
| --- | --- | --- |
| Performing the consultation with someone who was already familiar with their care (eg. their existing specialist melanoma nurse) | 10 (42%) | 14 (54%) |
| Using video during the consultation | 8 (33%) | 8 (31%) |
| Performing the consultation in person | 5 (21%) | 5 (19%) |
