# Supplementary material for: Feasibility, acceptability, and utility of a nurse-led survivorship program for people with metastatic melanoma (MELCARE)
Source: Support Care Cancer. 2022 Sep 22;30(11):9587–96. doi: 10.1007/s00520-022-07360-4 (PMC9492451; doi:10.1007/s00520-022-07360-4)
Supplement: Supplementary file 2 — Supplementary file2 (DOCX 21 KB) [file 520_2022_7360_MOESM2_ESM.docx]

**Supplementary text 1**

**MELCARE Additional Problem List**

|  | **Yes** | **No** |
| --- | --- | --- |
| 1. Rashes |  |  |
| 1. Vision problems |  |  |
| 1. Joint aches/ pains |  |  |
| 1. Muscle aches/ pains |  |  |
| 1. Fear of melanoma recurring or worsening |  |  |
| 1. Anxiety around the time of scans |  |  |
| 1. Information about screening for other cancers |  |  |
| 1. Information about vaccinations |  |  |
| 1. Information about skin checks |  |  |
| 1. Information about sun protection |  |  |
