# Supplementary material for: Feasibility, acceptability, and utility of a nurse-led survivorship program for people with metastatic melanoma (MELCARE)
Source: Support Care Cancer. 2022 Sep 22;30(11):9587–96. doi: 10.1007/s00520-022-07360-4 (PMC9492451; doi:10.1007/s00520-022-07360-4)
Supplement: Supplementary file 3 — Supplementary file3 (PDF 466 KB) [file 520_2022_7360_MOESM3_ESM.pdf]

		Issues	Explanation	Self management advice	Internet resources				Potential GP referrals
Physical	IO	Fatigue	Fatigue is excessive tiredness which is unlike the day-to-day tiredness a busy person may feel. Fatigue is very common among people who have received or are receiving immunotherapy or targeted therapy. Fatigue will usually continue while a person is receiving treatment, then gradually resolve once treatment is stopped. Learning how to cope with fatigue can improve your quality of life.	Here are some simple things you can do to reduce fatigue: Write down how you are feeling day to day. This can help you learn when you have the most and least energy. Plan a daily routine around how you are feeling. Pace yourself- attend to 1 thing at a time, include regular breaks and save your energy for when you need it most. It is important to remember not to overdo it during times that you have more energy. If you overdo it, you may be even more tired the next day. By physically active- discuss with your health care team what exercise is safe for your situation. Eat well, drink lots of water, and avoid alcohol and smoking.	This fact sheet from the Australian Cancer Survivorship Centre explains ways to cope with cancer and treatment-related fatigue. https://www.petermac.org/sites/default/files/ACSC_FactSheet_Survivor_Fatigue.pdf	The Cancel Council NSW website has information regarding the causes of fatigue and how to manage it. https://www.cancercouncil.com.au/cancer-information/managing-cancer-side-effects/fatigue/#Managing-fatigue	RESTORE is a free, online program designed to provide information about how you can self-manage fatigue using the most up-to-date evidence. This program was created by MacMillan Cancer Support and the University of Southampton in the United Kingdom. https://www.macmillanrestore.org.uk	The Cancer Council NSW has produced a podcast discussing cancer-related fatigue and how you can manage it. https://www.cancercouncil.com.au/podcasts/episode-3-managing-cancer-fatigue/	Talk to your GP to see whether any other causes of fatigue need to be checked, such as anaemia. Physiotherapy or exercise physiology- physiotherapists and exercise physiologists can help with improving your physical function, mobility and energy levels during and after immunotherapy or targeted therapy. Occupational therapy- occupational therapists can help you maximise your energy levels and improve your sense of wellbeing and quality of life. Social work- a social worker can put you in touch with organisations for home help such as house cleaning, meals or shopping to help you conserve your energy. These services are sometimes free through your local council. Talk to your GP about arranging any of these referrals.
		Sleep problems	Sleep issues are very common among people who have received or are receiving immunotherapy or targeted therapy. Sleep issues can occur while a person is receiving treatment, and then gradually improve once treatment is stopped. Sleep can also be affected by other health problems (eg. sleep apnoea) or medications (eg. steroids). Improving your sleep can improve your feeling of wellbeing.	Here are some simple things you can do to improve your sleep: Get up at the same time each morning. Exercise regularly during the day (not just before bed). Limit/ cut out alcohol, caffeine, nicotine and spicy food. Avoid daytime naps. Only go to bed when tired. Set a pre-sleep routine to help you relax. Avoid using electronic devices before bed or in the bedroom. Ensure the room is dark, quiet and at a comfortable temperature. If you wake up during the night, get up for a while before returning to bed.	The Cancer Council NSW website has information about managing sleep disturbance. https://www.cancercouncil.com.au/cancer-information/living-well/after-cancer-treatment/coping-with-side-effects-after-treatment/sleep-disturbance/	The Cancer Council NSW has produced a podcast discussing sleep and cancer and how you can improve your sleep quality. https://www.cancercouncil.com.au/podcasts/episode-12-sleep-cancer/			Talk to your GP to see whether any other medical causes of sleep disturbance need to be checked, such as sleep apnoea. Psychology- psychologists can provide cognitive behavioural therapy which has been shown to be effective in addressing sleep problems. Talk to your GP about arranging a referral.
		Rash/ dry skin	Skin problems, including rashes and dry skin, are common side effects of immunotherapy and targeted therapies. Rashes and dry skin will usually continue while a person is receiving treatment, then gradually resolve once treatment is stopped.	Please discuss with your medical oncology team if this is an ongoing issue for you. Your team may involve a dermatologist (skin specialist) in your care. Simple things you can do to look after your skin include: avoiding the sun and using sunscreen and wearing a hat when outside, using a soap free body wash, avoiding laundry detergents with strong perfumes, applying a hypoallergenic moisturiser to all skin after showering or bathing.	nil				nil
		Itch	Itch is a common side effect of immunotherapy. Itching will usually continue while a person is receiving treatment, then gradually resolve once treatment is stopped.	Please discuss with your medical oncology team if this is an ongoing issue for you. Your team may involve a dermatologist (skin specialist) in your care. Simple things you can do to reduce itching include: avoiding the sun and using sunscreen and wearing a hat when outside, using a soap free body wash, avoiding laundry detergents with strong perfumes, applying a hypoallergenic moisturiser to all skin after showering or bathing. Some people may find an anti-histamine useful to reduce itch.	nil				nil
		Joint aches/ pains	Joint aches and pains (arthralgias) are a common side effect of immunothearpy. Joint aches will usually continue while a person is receiving treatment, and may increase over time, then gradually resolve once treatment is stopped.	Please discuss with your medical oncology team if this is an ongoing issue for you. Your team may involve a rheumatologist (joint specialist) in your care. Simple analgesia (paracetamol, a few days of non-steroidal anti-inflammatory drugs) may be helpful.	nil				nil
		Diarrhoea	Diarrhoea is a common side effect of treatment. For people who have received or are receiving immunotherapy, this can be a sign of inflammation of the bowel (colitis). For people receiving targeted therapy, this could be a side effect of the treatment.	Please discuss with your medical oncology team as soon as possible if this is an ongoing issue for you. Tests will be needed to understand the cause of your diarrhoea and decide whether this is related to your treatment or another medical issue. Your team may involve a gastroenterologist (gut specialist) in your care.	nil				nil
		Shortness of breath	Shortness of breath is an uncommon side effect of immunotherapy. For people who have received or are receiving immunotherapy, this may be a sign of inflammation of the lungs (pneumonitis).	Please discuss with your medical oncology team as soon as possible if this is an ongoing issue for you. Tests will be needed to understand the cause of your shortness of breath and decide whether this is related to your treatment or another medical issue. Your team may involve a respiratory physician (lung doctor) or cardiologist (heart doctor) in your care.	nil				nil
		Dry mouth	Dry mouth is a common side effect of immunotherapy. Dry mouth will usually continue while a person is receiving treatment, and then gradually resolve once treatment is stopped.	Here are some simple things you can do to improve dry mouth: Use an alcohol-free mouthwash regularly to prevent infection. 1/2 tsp salt and/or 1 tsp bicarcbonate of soda in a glass of water is a good option. Sip fluids with meals and throughout the day. Limit alcohol and coffee as these can worsen dry mouth. Avoid smoking. Chewing sugar-free gum and sucking on ice cubes/ frozen pieces of fruit can stimulate the flow of saliva.	The Cancer Council NSW website contains information about managing dry mouth: https://www.cancercouncil.com.au/cancer-information/living-well/nutrition-and-cancer/treatment-side-effects-and-nutrition/dry-mouth/				Talk to your dentist about suitable mouth rinses and oral lubricants.
		Tingling in hands and feet	Tingling in the hands and feet (peripheral neuropathy) is a uncommon side effect of immunotherapy. It is important to understand the reason why you are experiencing tingling in the hands and feet, as this may be due to other medical issues (eg. diabetes). If it is due to immunotherapy, it may gradually improve over time once treatment has stopped.	Please discuss with your medical oncology team if this is an ongoing issue for you. Your team may involve a neurologist (nerve specialist) in your care.	The Cancer Council website contains information about managing peripheral neuropathy: https://www.cancer.org.au/cancer-information/cancer-side-effects/peripheral-neuropathy				Talk to your GP to see whether any other medical causes of tingling in the hands and feet need to be checked. Physiotherapy or exercise physiologist- a physiotherapist or exercise physiologist can develop an exercise program to help with walking and balance. Occupational therapist- an occupational therapist may suggest aids, equipment or strategies to manage your symptoms in your day to day life. Podiatrist- if the tingling affects your feet, a podiatrist can help look after your feet and check for injuries you may not have noticed. Talk to your GP about arranging any of these referrals.
	TT	Fevers	Fevers are a common side effect of some targeted therapies (eg. dabrafenib, encorafenib). They are usually most common during the first 3 months of treatment and then improve.	Please discuss this with your medical oncology team if this is an ongoing issue for you. Ways of managing the fevers may include briefly stopping the tablets until the fevers resolve, then restarting the tablets. If fevers continue despite this, small doses of steroid medication (prednisolone) may help to reduce fevers. Your doctor may also suggest taking the tablets on a less frequent basis.	nil				nil
		Rash	See immunotherapy rash						
		Blurred vision	Visual problems are an uncommon side effect of immunotherapy or targeted therapy. For people who have received or are receiving immunotherapy, this may be a sign of inflammation in the eye. For people receiving targeted therapy, this could be due to a problem at the back of the eye.	Please discuss with your medical oncology team as soon as possible if this an ongoing issue for you. Your team may involve an ophthalmologist (eye specialist) in your care.	nil				nil
		Nausea	Nausea is an uncommon side effect of immunotherapy or targeted therapy. For people who have received or are receiving immunotherapy, this may be a sign of inflammation in the stomach (gastritis). For people receiving targeted therapy, this could be a side effect of the treatment.	Please discuss with your medical oncology team as soon as possible if this an ongoing issue for you. Your team may involve a gastroenterologist (gut specialist) in your care. Here are some simple things you can do to improve nausea: take your anti-nausea medication as prescribed by your doctor, paying special attention to the timing of the doses. If these medications do not work, let your doctor know so they can offer you a different one to try. Eat small meals frequently (every 2-3 hours) during the day. Snack on bland/ dry foods (eg. crackers, toast, cereal). Eat and drink slowly, and chew your food well. Try drinks and food with ginger (eg. non-alcoholic ginger beer, ginger biscuits). Avoid foods that are sweet, fatty, fried, spicy or oily. Brush your teeth regularly and rinse your mouth to reduce unpleasant tastes that may worsen your nausea.	nil				nil
	Other	Lymphoedema	Many people experience lymphoedema due to surgery or radiotherapy for their melanoma. With early diagnosis, this condition can be very manageable.	Please discuss with your medical oncology team. They can help arrange a referral to your local lymphoedema therapist. Your lymphoedema specialist can fit you for compression clothing which can help reduce lymphoedema.	The Cancer Council's webiste contains information on lymphoedema and management https://www.cancercouncil.com.au/cancer-information/managing-cancer-side-effects/lymphoedema/treatment-and-management/	The Australasian Lymphology Association website has general information about managing lymphoedema. https://www.lymphoedema.org.au/about-lymphoedema/lymphoedema-management/#:~:text=Best%20practice%20management%20has%20a%20holistic%20multidisciplinary%20approach,Physical%20Therapy%20%28CPT%29%20or%20Complex%20Decongestive%20therapy%20%28CDT%29.			
		Poor memory/ concentration	Many people experience memory and concentration changes during their cancer journey. For some, the effects may be subtle, but for others they may be more obvious.	Please discuss with your medical oncology team if you have any issues with your memory or concentration.	The Cancer Council's website has useful links and resources in relation to cognition changes and cancer. https://www.cancercouncil.com.au/cancer-information/cancer-treatment/chemotherapy/side-effects/thinking-and-memory-changes/	Cancer Institute NSW's website has useful information on Cancer and Memory Changes https://www.cancer.nsw.gov.au/general-cancer-information/diagnosis-and-treatment/treatment/side-effects/memory-changes-chemo-brain			Talk to your GP about any changes in your thinking or ability to remember information. They can arrange referrals to an occupational therapist, who can assist you with strategies to to overcome specific challenges related to your concentration or memory. They can also refer you to a psychologist specialising in 'cognitive rehabilitation' to improve your symptoms by working on your attention, memory and navigation skills.
		Substance use		Please discuss with your medical oncology team if you have any issues with alcohol or substance use. They may refer you to see a doctor who specialises in dealing with alcohol and substance use.	The Reach Out website has resources regarding alcohol and substance use. https://au.reachout.com/mental-health-issues/addiction				

	Sexual dysfunction	Many patients encounter changes with their sexual intimacy following cancer treatment.	Please speak to your specialist melanoma nurse, who is trained to provide information on managing sexuality following cancer treatment. They may refer you to see a psychologist who has experience managing issues related to sexuality.	The Cancer Council has produced a helpful guide: 'Sexuality ,Intimacy and cancer'. https://www.cancer.org.au/assets/pdf/sexuality-intimacy-and-cancer-booklet				Talk to your GP about how you are feeling. Psychologist- a psychologist experienced in managing issues related to sexuality may be helpful. Your GP can assist in arranging this referral.
Psychological	Concerns regarding stopping treatment	Many people have concerns about stopping their treatment. This is a very normal concern to have. Some immunotherapy treatments may be stopped after 2 years if your melanoma is under control. This is a sign that you are likely to have long-term control of your melanoma. Many people are concerned that they will no longer have contact with their medical oncology team. However, you will continue to be followed up closely by your medical oncology team including regular scans to ensure the melanoma isn't coming back.	Please discuss this with your medical oncology team if this is an ongoing concern for you. They can explain to you why your treatment is stopping and how your melanoma will continue to be monitored.	The Cancer Council website has some general information about dealing with the different emotions people can feel while they are being treated for cancer. https://www.cancervic.org.au/living-with-cancer/emotions/getting-support.html	Melanoma Patients Australia offers a range of supports including peer support (via social media or telephone) and can arrange referrals to professional counselling and psychological support. https://melanomapatients.org.au/support/support-options/			Talk to your GP about how you are feeling. Psychologist- a psychologist specialisting in people with cancer may help you to discuss your feelings and suggest ways of managing these. This could include talking therapies (eg. cognitive behavioural therapies) or medication. Talk to your GP about arranging this referral.
	Concerns regarding long term side effects	Many people have concerns about the long-term side effects of their treatment. This is a very normal concern to have. Immunotherapy and targeted therapy may cause different long-term side effects. Common long-term side effects include fatgue, weight loss or gain, and changes in bowel behaviour. Managing these side effects can help improve your quality of life.	Please discuss with your medical oncology team if this is an ongoing issue for you. They may be able to provide more information, treatment or referrals depending on your specific concerns.	The Cancer Council website has general information about coping with side effects. https://www.cancervic.org.au/living-with-cancer/life-after-treatment/treatment-side-effects	The Melanoma Patients Australia (MPA) website has information about managing side effects on immunotherapy or targeted therapy. https://melanomapatients.org.au/lifestyle-risk-reduction/managing-side-effects/	Melanoma Patients Australia offers a range of supports including peer support (via social media or telephone) and can arrange referrals to professional counselling and psychological support. https://melanomapatients.org.au/support/support-options/		Seeing your GP regularly during treatment, and once treatment has finished, is important for maintaining your overall health and wellbeing. Your GP will continue to make sure you receive the right monitoring and treatment for general health conditions unrelated to your melanoma treatment.
	Scan-related anxiety	Many people report feeling anxious before, during or after having scans to check their melanoma. This is a very normal emotion to experience. However, there is a lot of support and advice that can be offered to help manage this emotion. This can help to improve how you feel around the time of scans.	Please discuss with your medical oncology team if this is an ongoing issue for you. They can help arrange information or referrals to help you manage anxiety around the time of scans.	Pink Hope, a breast cancer charity, has information on their website about scan-related anxiety. This is not specific to patients with melanoma. https://pinkhope.org.au/scans-anxiety-scanxiety/	The Patient Empowerment Network blog has advice about how you can manage scan-related anxiety. https://powerfulpatients.org/2018/07/24/coping-with-scanxiety-practical-tips-from-cancer-patients/	Melanoma Patients Australia offers a range of supports including peer support (via social media or telephone) and can arrange referrals to professional counselling and psychological support. https://melanomapatients.org.au/support/support-options/		Talk to your GP about how you are feeling. Psychologist- a psychologist specialisting in people with cancer may help you to discuss your anxiety about scans and suggest ways of managing these. Talking therapies, such as cognitive behavioural therapy, have been shown to improve scan-related anxiety. Talk to your GP about arranging this referral. Your GP can also create a Mental Health Care Plan which allows you to access Medicare-subsidised psychology sessions.
	Fear of cancer progression	Many people report concerns about their cancer worsening. This is a very normal emotion to experience, even if your cancer is well-controlled. People with a history of depression or anxiety may be at higher risk of experiencing these concerns.	Please discuss with your medical oncology team if this is an ongoing issue for you. Talking to your medical oncology team about your specific prognosis and signs of the melanoma coming back may help you understand your personal risk of the melanoma worsening or coming back. Attending support groups, telephone counselling or professional psychological supports may be helpful.	The Cancer Council NSW website has information regarding managing fear of cancer worsening. https://www.cancercouncil.com.au/cancer-information/living-well/after-cancer-treatment/fear-of-the-cancer-returning/managing-fear-of-recurrence/	Melanoma Patients Australia offers a range of supports including peer support (via social media or telephone) and can arrange referrals to professional counselling and psychological support. https://melanomapatients.org.au/support/support-options/	The Cancer Council NSW has produced a podcast discussing fear of cancer recurrence. https://www.cancercouncil.com.au/podcasts/episode-11-managing-fear/		Talk to your GP about how you are feeling. Psychologist- a psychologist specialisting in people with cancer may help you to discuss your concerns and suggest ways of managing these. Talking therapies, such as cognitive behavioural therapy, have been shown to help. Talk to your GP about arranging this referral. Your GP can also create a Mental Health Care Plan which allows you to access Medicare-subsidised psychology sessions.
	Depression	Many people report a low mood during or after treatment for their melanoma. This is a very normal emotion to experience. However, there is a lot of support and advice that can be offered to help improve your mood.	Please discuss with your medical oncology team if this is an ongoing issue for you. They can help arrange information or referrals to help you manage your concerns. Attending support groups, telephone counselling or professional psychological supports may be helpful. Two simple things that you can do for yourself to manage your mood are 1) exercise, which can boost your feelings of wellbeing and 2) 'behavioural activation'- this includes doing at least 1 activity for yourself a day and at least 1 'chore' to get it off your to-do list.	Melanoma Patients Australia offers a range of supports including peer support (via social media or telephone) and can arrange referrals to professional counselling and psychological support. https://melanomapatients.org.au/support/support-options/	Cancer.Net is a US website created by the American Society of Clinical Oncology (ASCO) that provides advice to cancer patients. This 6 minute podcast covers managing depression. https://www.cancer.net/sites/cancer.net/files/cancer_and_depression.mp3			Talk to your GP about how you are feeling. Psychologist- a psychologist specialisting in people with cancer may help you to discuss your concerns and suggest ways of managing these. Talking therapies, such as cognitive behavioural therapy, have been shown to help. Medications are sometimes also helpful. Talk to your GP about arranging this referral. Your GP can also create a Mental Health Care Plan which allows you to access Medicare-subsidised psychology sessions.
	Anxiety	Many people report feeling anxious during or after treatment for their melanoma. This is a very normal emotion to experience. However, there is a lot of support and advice that can be offered to help improve your mood.	Please discuss with your medical oncology team if this is an ongoing issue for you. They can help arrange information or referrals to help you manage your concerns. Attending support groups, telephone counselling or professional psychological supports may be helpful.	Melanoma Patients Australia offers a range of supports including peer support (via social media or telephone) and can arrange referrals to professional counselling and psychological support. https://melanomapatients.org.au/support/support-options/	Cancer.Net is a US website created by the American Society of Clinical Oncology (ASCO) that provides advice to cancer patients. This 6 minute podcast covers managing anxiety. https://www.cancer.net/sites/cancer.net/files/cancer_and_anxiety.mp3			Talk to your GP about how you are feeling. Psychologist- a psychologist specialisting in people with cancer may help you to discuss your concerns and suggest ways of managing these. Talking therapies, such as cognitive behavioural therapy, have been shown to help. Medications are sometimes also helpful. Talk to your GP about arranging this referral. Your GP can also create a Mental Health Care Plan which allows you to access Medicare-subsidised psychology sessions.
Social/ functional	Returning to work	Returning to work is a major step for people with cancer. You may have concerns around how to talk about your cancer with your employer, how to change your work to accommodate your treatment and its side effects.	The following resources may be helpful in understanding how to speak to your employer about restarting work, and how to plan for your return to work.	The Cancer Council Pro-Bono service can assist with helping you negotiating a return to work with your employer. Details about how to access this are available on the Cancer Council website: https://www.cancer.org.au/support-and-services/practical-and-financial-assistance/pro-bono-program	The Cancer Council NSW website has information about how to make a 'return to work plan'. https://www.cancercouncil.com.au/cancer-information/legal-work-and-financial-issues/work-and-cancer/for-workers/working-after-treatment-ends/returning-to-work/making-a-return-to-work-plan/	This fact sheet from the Australian Cancer Survivorship Centre answers common questions about returning to work or study. https://www.petermac.org/sites/default/files/ACSC_FactSheet_Survivor_Dealing_with_money_work_study.pdf	The Work after Cancer Project run by Flinders University provides information for people with cancer, their health care professionals, and employers regarding returning to work following a cancer diagnosis. https://www.workaftercancer.com.au/welcome/	Talk to your GP about your plans to return to work. Occupational therapy- an occupational therapist can help you make a plan to share with your employer about returning to work. Talk to your GP about arranging this referral.
	Financial assistance- paying with transport, parking, a	There can be many costs associated with cancer and its treatment. Concern, anxiety or worry about how to manage the financial impact of cancer is common. Knowing where to get support and the type of questions to ask can help survivors and their carers find a better quality of life.	If you would like know more about financial assistance, speak to your specialist melanoma nurse. They can provide advice, direct you to resources, or refer you to a Financial Counsellor through the Cancer Council who can provide more tailored advice, sometimes free of charge. The Cancer Council also offers small, once-off cash grants for urgent bills. If you need assistance with the cost of travelling to appointments or treatment, the Isolated Patients Travel and Accommodation Assistance Scheme (IPTAAS) may be able to assist. You can register for this online: http://www.iptaas.health.nsw.gov.au/For-patients/application-forms	The Cancer Council runs a Pro Bono Financial Assistance program, where people can access a financial counsellor sometimes free of charge. https://www.cancercouncil.com.au/get-support/pro-bono-program/	The Cancer Council website has general information about managing your finances following a cancer diagnosis. https://www.cancercouncil.com.au/cancer-information/legal-work-and-financial-issues/finances/			
	Accessing super	Many people with cancer have concerns about their superannuation, including whether they are entitled to access it early.	If you would like know more about accessing super, speak to your specialist melanoma nurse. They can provide general advice, write letters of support or complete paperwork for your superannuation fund, or refer you to see a Financial Counsellor through the Cancer Council who can provide more tailored advice, sometimes free of charge. You can also speak to your superannuation fund directly about how you can access your superannuation.	The Cancer Council NSW website has specific information regarding superannuation. https://www.cancercouncil.com.au/wp-content/uploads/2020/04/Super-and-Cancer_NSW.pdf	The Cancer Council runs a Pro Bono Financial Assistance program, where people can access a financial planner sometimes free of charge. https://www.cancercouncil.com.au/get-support/pro-bono-program/			
	Life insurance	Life insurance is insurance paid as a lump sum, as an income stream (to a nominated beneficiary), or as a combination of the two. Some policies will pay the insured amount if you are diagnosed with a terminal medical condition. Many people with cancer have concerns about their life insurance and whether they are able to make a claim.	Many people don't realise they have life insurance attached to their superannuation. Many industry super funds, and some retail funds, offer insurance by default. In many cases, you will be covered unless you chose to 'opt out'. If you would like know more about your life insurance, speak to your specialist melanoma nurse. They can provide general advice, write letters of support or complete paperwork for your insurer, or refer you to see a Financial Counsellor through the Cancer Council who can provide more tailored advice, sometimes free of charge. You can also speak to your insurer directly about whether you can make a claim. People often don't realise that they may have insurance attached to their super.	The Cancer Council NSW website has specific information about life insurance. https://www.cancercouncil.com.au/cancer-information/legal-work-and-financial-issues/finances/finding-other-income/#insurance-claims	The Cancer Council runs a Pro Bono Financial Assistance program, where people can access a financial planner sometimes free of charge. https://www.cancercouncil.com.au/get-support/pro-bono-program/			
	Discussing melanoma with children	Speaking to your children about your melanoma may be challenging. For people with stage 4 melanoma, it can be difficult to explain your diagnosis, treatment, and how the melanoma is likely to affect you in the future in a way that your children are able to understand.	If you would like assistance talking about your melanoma with children, your specialist melanoma nurse may be able to provide guidance and advice. Please talk to your medical oncology team if this would be helpful to you.	The Cancer Council's 'Talking to Kids about Cancer' booklet has information about how to discuss your melanoma with your children. https://www.cancercouncil.com.au/cancer-information/for-family-and-friends/talking-to-kids-about-cancer/	Melanoma Institute of Australia has produced a children's comic explaining the basics about melanoma. It can be accessed for free here: https://www.jumohealth.com/comics/understanding-melanoma-7-12-digital-en	Canteen is an organisation dedicated to helping young adults (12-25 years old) cope with cancer, including a cancer diagnosis in their family. For more information, please see the Canteen website: https://www.canteen.org.au/young-people/my-parent-has-cancer/		nil
	Discussing melanoma with family	Speaking to family and friends about your melanoma may be challenging. For people with stage 4 melanoma, it can be difficult to explain your diagnosis, treatment, and how the melanoma is likely to affect you in the future.	If you would like assistance talking about your melanoma with friends and family, your specialist melanoma nurse can speak to family on your behalf to answer questions. Please talk to your medical oncology team if this would be helpful to you.	The Cancer Council booklet 'Understanding melanoma' has information about how to discuss your melanoma with your family and friends. https://www.cancercouncil.com.au/wp-content/uploads/2014/05/Understanding-Melanoma-2021.pdf	The Cancer Council also produces a helpful booklet for family and friends caring for someone with cancer. https://www.cancer.org.au/assets/pdf/caring-for-someone-with-cancer-booklet#_ga=2.220541848.1218782961.1623042910-447758124.1604549587			nil
	Fertility	The effects of immunotherapy and targeted therapy on fertility are not yet fully understood. Studies so far suggest that immunotherapy may not affect fertility in men or women. However, more research is underway to understand these effects better.	Please discuss with your medical oncology team if you have any questions about fertility. Your team may involve a fertility specialist in your care if further testing is required.	nil				nil

**Sun exposure**	Future sun exposure/ adherence to sun protection m	It is important to continue to protect your skin from the sun after you have been diagnosed with melanoma.	Follow SunSmart behaviour (slip on sun-protective clothing, slop on SPF30 broad spectrum, water resistant sunscreen at least 20 minutes before going outdoors and re-apply every 2 hours, slap on a broad brimmed hat, seek shade, slide on sunglasses). Use the SunSmart UV Alert to check the recommended sun protection times in your local area every day (available as an app, online, in the weather section of the newspaper). Some people may be concerned that they are not getting enough vitamin D. The amount of sunlight you need for vitamin D depends on several factors including the UV level, your skin type and lifestyle. For most people, just 15-20 minutes of incidental sun exposure is enough to produce the required vitamin D level. If you are concerned about vitamin D deficiency, talk to your GP about the best ways to maintain vitamin D while reducing your risk of further melanomas.	For more information about sun protection following a melanoma diagnosis, please see the Cancer Council website: https://www.cancervic.org.au/cancer-information/types-of-cancer/melanoma/life-after-melanoma.html	nil
	Familial melanoma risk	Following a diagnosis of melanoma, some people may be concerned about the risk of melanoma in their family members. A small number of families have a greater risk of melanoma because they have inherited a change in a specific gene which causes melanoma.	If you have a personal history of melanoma at an early age, more 1 skin melanoma, a large number of moles (>10 on the arms, >200 on the body), multiple unusual or atypical moles (moles with an irregular border, slight variation in colour or asymmetry), melanoma on areas of the body not exposed to the sun, or a family history of melanoma, there may a risk of familial melanoma. Other concerning factors include multiple cases of melanoma on the same side of the family, ocular (eye) melanoma, pancreatic or breast cancer in more than 1 family member, or several relatives with the same type of cancer. Talk to your medical oncology team to decide whether genetic testing is required. If testing is required, your team will refer you to a Familial Cancer Centre in your area.	For more information about the risk of familial (inherited) melanoma, please see the Cancer Council website: https://www.cancervic.org.au/cancer-information/genetics-and-risk/familial-melanoma	
**Secondary prevention**	Skin checks	It is important to continue skin checks to monitor for any new melanomas. We recommend having a skin check performed at least once a year by your GP, dermatologist, or melanoma surgeon.	Get to know your skin and be aware of what looks normal for you so that you can find changes earlier. Check all your skin (including non-sun exposed areas) on a regular basis. If you notice anything unusual, including a change in shape, size, colour of a spot or new spot, bring this to the attention of your GP and dermatologist as soon as possible.	Nil	If you would prefer your skin checks to be carried out by a dermatolgist or melanoma surgeon, talk to your GP about arranging a referral.
	Screening for secondary malignancies	It is important to continue screening for other cancers, even after you have been treated (or are currently being treated for) cancer. There are 3 cancer screening programs in Australia for breast, cervical and bowel cancer.	Talk to your GP to arrange your screening tests. Your GP can advise what screening tests are needed for your age and gender.	For more information on cancer screening in Australia, please see the Cancer Council website: https://www.cancer.org.au/cancer-information/causes-and-prevention/early-detection-and-screening	nil
	Vaccinations	It is important to continue with immunisations according to your age group. This includes vaccinations against COVID-19, influenza and pneumonia.	Talk to your GP about what vaccinations you need for your age group. If you are on a clinical trial, please speak to your clinical trials team BEFORE having any vaccinations.	For more information about what vaccinations you should have for your age group, please see the Australian Government's National Immunisation Program website. https://www.health.gov.au/health-topics/immunisation/immunisation-throughout-life/national-immunisation-program-schedule	
