# Supplementary material for: Feasibility, acceptability, and utility of a nurse-led survivorship program for people with metastatic melanoma (MELCARE)
Source: Support Care Cancer. 2022 Sep 22;30(11):9587–96. doi: 10.1007/s00520-022-07360-4 (PMC9492451; doi:10.1007/s00520-022-07360-4)
Supplement: Supplementary file 4 — Supplementary file4 (PDF 153 KB) [file 520_2022_7360_MOESM4_ESM.pdf]

Melanoma Institute Australia   mpa MELANOMA PATIENTS AUSTRALIA   PoCoG Psycho-oncology Co-operative Research Group

Your Survivorship Care Plan

This is a brief record of your recent cancer treatment and your goals and priorities for your care.   You can share this with anyone involved in your care.

Personal details			
**Name**:		**DOB:**	
**Medical Oncologist**:		Melanoma Institute Australia, Sydney Medical Oncology, Phone: 02 9911 7258	
**Melanoma nurse consultant:**			

Treatment Summary	
**Diagnosis**:	
**Most recent drug treatment**:	
**Current status of melanoma:**	Currently well-controlled with likely long-term disease control

Your priorities

	Issue	General information	How you can manage this at home	Helpful websites and podcasts	Referral suggestions	Comments
1		#N/A	#N/A	#N/A<br>#N/A<br>#N/A<br>#N/A	#N/A	
2		#N/A	#N/A	#N/A<br>#N/A<br>#N/A<br>#N/A	#N/A	

3		#N/A	#N/A	#N/A<br>#N/A<br>#N/A<br>#N/A	#N/A	

General advice			
	**General information**	**How can I manage this at home?**	**Helpful websites and podcasts**
**Skin checks**	It is important to continue skin checks to monitor for any new melanomas. We recommend having a skin check at least once a year by your GP, dermatologist, or melanoma surgeon.	- Get to know your skin and be aware of what looks normal for you so that you can find changes earlier.<br>- Check all your skin (including non-sun exposed areas) on a regular basis.<br>- If you notice anything unusual, including a change in shape, size, colour of a spot or new spot, bring this to the attention of your skin check doctor as soon as possible.	For more information about skin checks, please see the Melanoma Institute Australia website. bit.ly/MELcheck
**Sun protection**	It is important to continue to protect your skin from the sun after you have been diagnosed with melanoma. New melanomas on the skin can still occur while you are being treated with (or have previously been treated with) immunotherapy or targeted therapy.	- Follow SunSmart behaviour- slip on sun-protective clothing, slop on SPF30 broad spectrum, water resistant sunscreen at least 20 minutes before going outdoors and re-apply every 2 hours, slap on a broad brimmed hat, seek shade, slide on sunglasses.<br>- Use the SunSmart UV Alert to check the recommended sun protection times in your local area every day (available as an app, online, in the weather section of the newspaper).<br>- Some people may be concerned that they are not getting enough vitamin D. The amount of sunlight you need for vitamin D depends on several factors including the UV level, your skin type and lifestyle. For most people, just 15-20 minutes of incidental sun exposure is enough to produce the required vitamin D level. If you are concerned about vitamin D deficiency, talk to your GP about the best ways to maintain vitamin D while reducing your risk of further melanomas.	For more information about sun protection following a melanoma diagnosis, please see the Cancer Council website. bit.ly/CVsun
**Screening for other cancers**	It is important to continue screening for other cancers, even after you have been diagnosed with melanoma. There are 3 cancer screening programs in Australia: breast, cervical and bowel cancer.	- Talk to your GP to arrange your screening tests. Your GP can advise what screening tests are needed for your age and gender.	For more information on cancer screening in Australia, please see the Cancer Council website. bit.ly/CAUscreening
**Vaccinations**	It is important to continue with immunisations according to your age group. This includes vaccinations against COVID-19, influenza, pneumonia and shingles.	- Talk to your GP about what vaccinations you need for your age group. If you are on a clinical trial, please speak to your clinical trials team BEFORE having any vaccinations.	For more information about what vaccinations you need, please see the Australian Government's National Immunisation Program website. https://bit.ly/NIPschedule

If you have any questions regarding this plan, please contact Ms Brooke Kelly (brooke@melanomapatients.org.au).

Prepared by: Brooke Kelly, Registered Oncology Nurse (Div 1) **Date:**
