# Supplementary material for: Feasibility, acceptability, and utility of a nurse-led survivorship program for people with metastatic melanoma (MELCARE)
Source: Support Care Cancer. 2022 Sep 22;30(11):9587–96. doi: 10.1007/s00520-022-07360-4 (PMC9492451; doi:10.1007/s00520-022-07360-4)
Supplement: Supplementary file 5 — Supplementary file5 (DOCX 35 KB) [file 520_2022_7360_MOESM5_ESM.docx]

**MELCARE Participant Survey**

**Please select the appropriate response.**

**Part 1: Initial appointment**

1. *How helpful did you find the initial appointment?*
2. Not helpful at all
3. Not helpful
4. Neutral
5. Helpful
6. Very helpful
7. *What did you find most helpful about the initial appointment? (tick all that apply)*

- Discussing my side effects
- Being given advice about how to manage my side effects
- Discussing how I’m feeling emotionally
- Being given advice about how to manage my mood and emotions
- Discussing practical issues, such as work and finances
- Being given advice about how to manage practical issues, such as work and finances
- Setting personal goals for my care
- Being provided with a written care plan to show my GP and other health care professionals
- The initial appointment was not helpful
- Other (free text)

1. *Was there enough time to discuss issues important to you?*

- Yes
- No

If no, how much extra time would have been helpful?

- <10 mins
- 10-20 mins
- >20 mins

1. *How would you improve the initial appointment?*

- Make it longer
- Do it in person
- Do it with someone who is already familiar with my care eg. my specialist melanoma nurse from Melanoma Institute Australia
- Do it with video eg. Via Zoom
- Other (free text)

**Part 2: Survivorship care plan**

1. *Did you read your survivorship care plan?*

- Yes
- No

1. *If yes, how easy was it to read and understand?*
2. Very difficult to understand
3. Difficult to understand
4. Neutral
5. Easy to understand
6. Very easy to understand
7. *Have you discussed your survivorship care plan with your general practitioner?*

- Yes
- No

1. *If not, are you planning to discuss your survivorship care plan with your general practitioner in the next 3 months?*

- Yes
- No

*If no, why?*

- I would prefer to discuss this with my usual cancer care team
- I don’t have time
- I don’t think my general practitioner will be able to help me
- I don’t need any help from my general practitioner
- I cannot afford to visit my general practitioner
- Other (free text)

1. *What aspect of the survivorship care plan was most useful? (tick all that apply)*

- Setting personal goals for my ongoing care
- Being provided with written advice about how I can manage my health
- Being provided with websites and podcast recommendations
- Being provided with suggestions for referrals to other health care professionals
- General information regarding vaccinations
- General information regarding skin checks
- General information regarding checking for other cancers
- Having a written document to share with my health care team (eg. General practitioners, allied health professionals such as physiotherapists, occupational therapists, psychologists)
- The survivorship care plan was not helpful
- Other (free text)

1. *What could be improved about the survivorship care plan? (tick all that apply)*

- It could provide more detail about:
  - My personal melanoma diagnosis and treatment
  - How I can manage my health
  - Internet resources
  - Potential referrals that my general practitioner can arrange
- The plan was not specific enough to my issues/ needs
- The advice provided in the care plan was too general in nature
- Other (free text)

**Part 3: Follow up appointment**

1. *How helpful did you find the follow-up appointment?*
2. Not helpful at all
3. Not helpful
4. Neutral
5. Helpful
6. Very helpful
7. *Was there enough time in the follow-up appointment to revisit any issues raised in your initial appointment?*

- Yes
- No

*If no, how much more time would have been helpful?*

- <10 mins
- 10-20 mins
- >20 mins

1. *What did you find most helpful about the follow-up appointment?*

- Discussing my side effects
- Being given advice about how to manage my side effects
- Discussing how I’m feeling emotionally
- Being given advice about how to manage my mood and emotions
- Discussing practical issues, such as work and finances
- Being given advice about how to manage practical issues, such as work and finances
- Reviewing my personal goals for my care, and how I have managed to work towards these since the initial appointment
- Discussing ways I can get ongoing support from Melanoma Patients Australia once the study ends
- The follow-up appointment was not helpful
- Other (free text)

1. *How would you improve the follow up appointment?*

- Make the appointment longer
- Conduct the appointment in person
- Conduct the appointment with someone who is already familiar with my care eg. my specialist melanoma nurse from Melanoma Institute Australia
- Conduct the appointment with video eg. Via Zoom
- Other (free text)

1. *Did you want to have more contact with the telehealth nurse, or did you feel that 2 appointments was enough?*

- 1 appointment was enough
- 2 appointments was enough
- More than 2 appointments are needed

**Part 4: Overall Impressions of MELCARE**

*The MELCARE program meets my approval.*

Completely agree

Agree

Neither agree nor disagree

Disagree

Completely disagree

*The MELCARE program is appealing to me.*

Completely agree

Agree

Neither agree nor disagree

Disagree

Completely disagree

*I like the MELCARE program.*

Completely agree

Agree

Neither agree nor disagree

Disagree

Completely disagree

*I welcome the MELCARE program as part of my care.*

Completely agree

Agree

Neither agree nor disagree

Disagree

Completely disagree

1. *Did the MELCARE program improve your overall satisfaction with your melanoma care?*

- Yes
- No

1. *Would you recommend the MELCARE program to other people with melanoma?*

- Yes
- No

1. *Did the MELCARE program help you manage issues that were not discussed during your medical oncology appointments?*

- Yes
- No

If yes, which ones?

Side effects from my treatment

Emotional/ mood issues

How to discuss my diagnosis with family

How to discuss my diagnosis with my children

Practical issues eg. Returning to work, finances

Strategies for protecting myself from the sun

Information regarding skin checks

Ways in which I can be monitored for other cancers

Information about what vaccinations I should receive

- Other (free text)

**THANK YOU FOR COMPLETING THIS SURVEY**

Your time and thoughts are highly valued.
